# Dental Trauma in Children with Autistic Disorder: A Retrospective Study

**DOI:** 10.1155/2021/3125251

**Published:** 2021-09-08

**Authors:** Paola Martina Marra, Stefano Parascandolo, Luca Fiorillo, Marco Cicciù, Gabriele Cervino, Cesare D'Amico, Rosa De Stefano, Paola Salerno, Umberto Esposito, Annalisa Itro

**Affiliations:** ^1^Complex Operative Unit of Stomatological Surgery in Developmental Age, University of Campania Luigi Vanvitelli, Naples, Italy; ^2^Complex Operative Unit of Odontostomatology, A. Cardarelli General Hospital, Naples, Italy; ^3^Department of Biomedical and Dental Sciences and Morphofunctional Imaging, School of Dentistry, Messina University, Messina, Italy; ^4^Department of Biomedical and Dental Sciences and Morphofunctional Imaging, Messina University, Messina, Italy

## Abstract

**Background:**

The oral health care of autistic children is elaborated; they often fail to define dental problems, and a family-centered approach can be useful to improve and intercept these disorders.

**Aim:**

To assess the oral status of autistic children, comparing it with no autistic patients.

**Materials and Methods:**

A retrospective study analyzed the oral health status of 70 children, 35 with autism and 35 without the disorder. Conditions assessed were dental trauma type, periodontal tissue injuries, soft tissue lip injuries, different treatments carried out, associated soft tissue findings and disorders, and the long-term management. All patients (≤15 years of age) were chosen consecutively.

**Results:**

Females (57%) suffered more traumatic injuries than males (43%) in the autistic group, whereas males affected by dental trauma (54%) are predominant in the control group. The enamel fracture was the main finding among the dental trauma types in both groups followed by enamel/dentin/pulp fracture (31%), root fracture (11%), and avulsions (3%) in the autistic group and by avulsions (20%), root fracture (11%), and enamel/dentin/pulp fracture (6%) in the control group. The comparison of all variables of the two groups showed a statistically significant difference (*P* < 0.012). The lower lip was statistically more injured than the upper lip (*P* < 0.005).

**Conclusions:**

The composite restorative technique was the most common approach carried out; the long-term evaluation, when possible, was predominantly managed through root canal therapy in the control group (81%), and root canal therapy (50%) and tooth extraction (50%) in the sample group.

## 1. Introduction

Autism or autistic disorder is a severe developmental disability that is characterized by an impairment in mutual social interactions, communication skills, and repetitive patterns of behaviors. It is the third most frequent disorder after mental retardation and cerebral palsy and it was described by Kanner in 1943 for the first time [1].

A severe abnormality of reciprocal social relatedness and of communication development, frequently including spoken language, repetitive and stereotypical patterns of conduct, and a premature onset (before the age of 3 years) are four criteria that allow the diagnosis of autism concurrently with neurological, psychological, and medical examinations. The incidence among males is 3 to 4 times higher than females [2, 3].

Many doubts concern the possible causes. The postencephalitic infection, genetic autoimmune or environmental factors, and vitamin D deficiency are described as some of the possible aetiological factors [4, 5]. Antidepressants, valproic acid, and thalidomide during pregnancy and maternal viral infections are also reported in the literature [4–6].

A prevalence of 1.5% in developed countries is described [6].

Autistic children frequently show severe behavioral disturbances such as aggression and self-injuries [7, 8] that often impede the rehabilitative efforts and represent a huge challenge to the parents. They can also show an increased sensitivity to sounds, light, odours, and colours.

A traumatic dental trauma can represent a substantial dentofacial and general health problem that may have medical, esthetic, and psychological consequences considering that some of the main medical conditions described are anxiety, depression, and seizures [5–11].

Some papers reported that malocclusions are directly proportional to the severity of mental disabilities [9–14]. Since these patients tend to present an anterior open bite with incisor flaring and class II malocclusion, they are more subjected to suffer dental trauma, specifically involving the central and lateral upper incisors [5–11].

Inside this context, a more focused screening (clinical checkup, pretreatment radiographs, etc.) and precautionary interventions (e.g., reducing the increased overjet) should be aimed at children with class II dentoskeletal malocclusion using specific orthodontic appliances [13, 14]. Although the orthodontic treatment represents a challenge for clinicians due to the reduced patient cooperation, in the literature, these types of approaches are described [15].

To date, very few and dated papers have reported the association between dental trauma and hard and soft tissue injuries.

Borzabadi-Farahani et al. [14] explored the association between maxillary incisor trauma and facial skeletal vertical pattern, overjet, lips, and gender in five hundred and two subjects.

They found that subjects with competent lips and overjet of more than 3.5 mm suffered more upper incisor trauma.

Since the prevalence of this disorder has increased over the years, we think that it is very important to know how to handle these dental emergencies in development age considering also that no studies assessed the relationship between the maxillary incisor trauma and dental, periodontal, and soft tissue injuries in autistic children comparing it with the no autistic group.

Therefore, the aim of the present study was to investigate the prevalence and the frequency of different kinds of trauma, injured lips, treatments, associated lesions and disorders, and long-term management in the autistic and no autistic patients.

## 2. Materials and Methods

### 2.1. Study Sample

After obtaining the ethical approval (n. 16947/2020) and the informed consents by the parents' patients, the diagnosis of dental trauma concerning the upper central and lateral incisors was retrieved from the database of both the Complex Operative Unit of Stomatological Surgery in Developmental Age of University of Campania Luigi Vanvitelli and the Complex Operative Unit of Odontostomatology of A. Cardarelli General Hospital in Naples.

This retrospective study analyzed data regarding the oral health status of 70 patients assembled from January 2016 to January 2020. All patients (≤15 years of age) were chosen consecutively. Of the 70 initial patients, the sample group was constituted by 35 autistic children (15 males with a mean age of 11.2 and 20 females with a mean age of 12.6), whereas 35 patients without the autistic spectrum represented the control group (19 males with a mean age of 10.5 and 16 females with a mean age of 9).

For each patient of both groups, the following parameters were collected:

- Dental trauma type (enamel fracture, enamel/dentin/pulp fracture, and root fracture)
- Periodontal tissue injuries (avulsion)
- Soft tissue lip injuries (upper and/or lower lip)
- Soft tissue lip injury types (erosion and/or ulceration)
- Different treatments carried out (composite restorative technique and/or root canal therapy, checkup, and tooth extraction)
- Associated soft tissue findings (chin erosion and finger biting)
- Associated disorders (epilepsy and attention-deficit/hyperactivity disorder or ADHD)
- The long-term management (<1 year)

One examiner grouped all data regarding the diagnosis of dental trauma from the hospitals' database.

The exclusion criteria were trauma injuring different teeth, syndromes, and craniofacial anomalies.

## 3. Statistical Analysis

Data was entered into a computer using patient identification number; the distribution of each variable was calculated in percentage. Wilcoxon test, Mann-Whitney test, and Student's *t*-test were used to compare quantitative variables, and the chi-squared (*z*) test was used to compare qualitative variables. A *P* value smaller than 0.050 was considered to be statistically significant.

## 4. Results

Table 1 shows the age and sex distribution of the sample and control groups. In our findings, females (57%) suffered more traumatic injuries than males (43%) in the autistic group, whereas dental trauma affecting males (54%) are predominant in the control group.

Table 2 summarizes the distribution for both groups of the different dental trauma types, periodontal tissue injuries, soft tissue lip injuries and types, different treatments carried out, associated soft tissue findings, associated disorders, and the long-term management.

As regards the dental trauma types, the most frequent encountered injuries were the enamel fractures in both the autistic and control groups, representing the 54% and 63%, respectively. In the autistic group, we found an occurrence of 31% of enamel/dentin/pulp fracture, 11% of root fracture, and 3% of avulsions.

In the control group, instead, the enamel fractures were followed by a 20% of avulsions, 11% of root fracture, and 6% of enamel/dentin/pulp fracture. The comparison of all variables of the two groups showed a statistically significant difference (*P* < 0.012).

Regarding the soft tissue lip injury location, the only lower lip was involved a 100% of times in the autistic group and an 80% of times in the control group, showing a statistically significant difference (*P* < 0.005).

**Figure 1:**
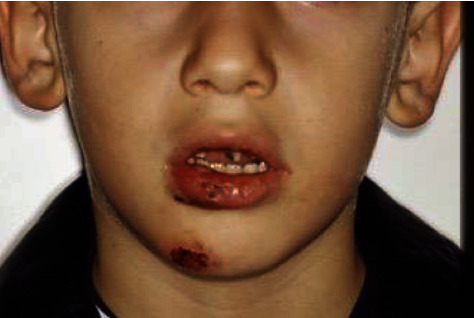
Frontal view of 8-year-old male suffering dental trauma correlated to chin erosion and ulceration and lower lip injuries.

As regards the types of soft tissue lip injuries, both the erosions and ulcerations were reported (Figure 1).

Regarding the treatments, the most recurrent managements of dental trauma among the autistic patients were in order as follows: composite restorative technique (34%), composite restorative technique and root canal therapy (26%), only observation (23%), and tooth extractions (17%), while in no autistic patients were as follows: composite restorative technique (49%), only observation (37%), tooth extractions (9%), and composite restorative technique and root canal therapy (6%) (*P* < 0.057).

The chin erosion was found in the 85% of the autistic patients and in the 83% of no autistic patients followed by the finger biting in the 5% in the autistic patients and 17% in no autistic patients.

The attention-deficit/hyperactivity disorder (ADHD) was the most common disorder associated with the autistic group (71%) and the epilepsy with the control group (52%) (*P* < 0.089).

Finally, our data regarding the long-term approach included the root canal therapy after few years and the extractions; in the autistic patients, we found a 50% of both approaches, while in no autistic patients, the most prevalent treatment was the root canal therapy (81%).

## 5. Discussion

To our knowledge, very few and dated papers investigated the incidence and prevalence of dental trauma among autistic children and even less articles comparing the oral injuries in these patients with a control group [16–22].

In our findings, females (57%) suffered more traumatic injuries than males (43%) in the autistic group, whereas dental trauma affecting males (54%) are predominant in the control group (Table 1). Our results are in contrast with many authors who found a greater prevalence of autistic males with incisor traumatic injuries [12–14, 20–22], although there are other studies showing an equivalent distribution in both sexes [23, 24].

The literature reports that autistic children with primary or mixed dentition can present higher quantities of dental caries and gingivitis while children with permanent dentition had significantly more gingivitis and malocclusion when compared to other oral disorders [14].

In our opinion, since the increasing occurrence of the autistic disorder [6], it is very important for the clinicians to know how to manage these dental injuries affecting patients in developmental age, ensuring an adequate and minimally invasive management using a prompt approach, when possible.

Some epidemiological researches reported a higher prevalence of trauma affecting the upper incisors [21]; enamel fractures followed by the fractures of the enamel and the dentine have been described as the most frequent oral disorders [22–26].

Our data partly agree with the authors' findings, since we also found a bigger prevalence of enamel fracture in both groups, but the fractures including the enamel, dentin, and pulp were our second most frequent discovery, particularly in the autistic patients (31%).

The most prevalent dental trauma type in Iranian children was the fracture of enamel only, representing 69% of injured teeth [13]. Moreover, the fracture involving enamel, dentin, and pulp affected 8.8% of the fractured maxillary incisors in children without autism [13].

The muscular incoordination of autistic patients is described as a comorbidity that can provoke accidental falls, producing dental trauma [26–30]. Moreover, this altered muscle tonus often results in an open bite with labial flaring of the maxillary incisors and lip incompetence, predisposing these teeth to fractures. In addition, some authors reported a significant association between class II skeletal pattern and maxillary incisor trauma [12–14]. Our data are consistent with them [12–14]; most of the patients presented a prominent upper maxilla with a dental class II division I malocclusion, as registered in the computerized medical records, and some of them have damaged their upper incisors after a fall; the higher incidence found on enamel fracture (54%), the erosions and ulcerations of the lower lip (94%), and the chin erosions (95%) can be the demonstration (Figure 1).

In general, there is a high risk of oral injuries during childhood and adolescence. The control group, in our study, also shows a bigger prevalence of enamel fracture (63%) treated with a composite restorative technique, and when it was not possible, due to the lack of cooperation (48%), the patients were monitored through only observation and a routine checkup (37%).

It is habitually arduous to offer to hyperactive or epileptic patients dental treatments; they usually take medications and show lack of communication and hostile behaviors. Even if the composite restorative approach was the most common finding, the extraction was usually the only possible option, especially in the autistic children.

The surgical approach, especially if carried out too early, can lead to other dental problems, such as loss of space in the dental arches, incorrect space closure, and dental overeruption, which may not be easily solved. So, a good communication can help to establish trust and build needed cooperation throughout the visit and treatment.

All patients in developmental age, especially with health disorders, need experienced doctors who know how to face promptly dental trauma under general anesthesia or in the dental chair, if possible.

Both the Unit of Complex Operative Unit of Stomatological Surgery in Developmental Age of University of Campania Luigi Vanvitelli and the Complex Operative Unit of Odontostomatology of A. Cardarelli General Hospital have specific medical units that deal with the uncooperative patients to carry out all health needs.

In both the sample and control groups, respectively, 3% and 20%, there is very little incidence of periodontal tissue injuries, like the avulsion, maybe because the alveolar bone is more compact and more resistant to lateral and axial movements in mixed dentition.

If not promptly treated, the dental hard tissue and pulp injuries and periodontal diseases can provoke several complications, as the external root resorption, surface or replacement resorption, and ankylosis [28, 29].

In our opinion, the small number of patients with the autistic spectrum registered who suffered tooth avulsion, over the 4 years, testifies how these patients are constantly monitored and followed by their families and specialized centers.

This study has some limitations: the limited number of patients, the recruitment of patients was consecutive, and no randomization was performed.

An additional limitation is that we considered a younger population (≤15 years of age); our data could not be applied to the older people since younger patients should have more dental trauma risk factors; thus, our findings could not be certainly appropriate to the general population.

More epidemiological researches in dental trauma centered are needed from representative samples using standardized trauma classifications to set up a baseline for future preventive and management plans, and prospective randomized controlled trials are required to evaluate the behavioral variables in reducing the occurrence of incisor trauma.

Despite the retrospective nature of the study, fortunately, it was possible to investigate and record the main oral injuries, thanks to the computerization of medical records.

## 6. Conclusion

The dental trauma variation statistically differs among the autistic and the control groups (*P* < 0.012), the lower lip was the most injured soft tissue involved (*P* < 0.005), the composite restorative technique was the most common treatment carried out, and the long-term approach, when possible, was managed through root canal therapy in the control group (81%) and through root canal therapy (50%) and extraction (50%) in the sample group.

## Figures and Tables

**Table 1:** Demographics of the sample group (autistic children) and the control group (nonautistic children).

	Sex	*N*	Age (year) Mean ± SD
Sample group	Males	15	11.2 ± 2
	Females	20	11.8 ± 1.5

Control group	Males	19	10.5 ± 3.5
	Females	16	9 ± 2

**Table 2:** Distribution of the different kinds of trauma, affected lips with their prevalent lesions, treatments, associated lesions and disorders, and long-term management in the sample and the control group.

	Autistic group	Control group	*P* value
*Dental trauma type*			
Enamel fracture	19 (54%)	22 (63%)	<0.012^∗^
Enamel/dentin/pulp fracture	11 (31%)	2 (6%)	
Root fracture	4 (11%)	4 (11%)	
Avulsion	1 (3%)	7 (20%)	

*Soft tissue lip injury location*			
Upper lip and lower lip	0 (0%)	7 (20%)	<0.005^∗^
Lower lip	35 (100%)	28 (80%)	

*Soft tissue lip injury type*			
Erosion and ulcerations	33 (94%)	33 (94%)	
Erosion	2 (6%)	2 (6%)	

*Treatments*			
Composite restorative technique	12 (34%)	17 (49%)	<0.057
Composite restorative technique and root canal therapy	9 (26%)	2 (6%)	
Checkup	8 (23%)	13 (37%)	
Tooth extraction	6 (17%)	3 (9%)	

*Associated soft tissue findings*			
Chin erosion	19 (95%)	24 (83%)	<0.20
Finger biting	1 (5%)	5 (17%)	

*Associated disorders*			
Epilepsy	9 (29%)	11 (52%)	<0.089
Attention-deficit/hyperactivity disorder (ADHD)	22 (71%)	10 (48%)	

*Long-term management*			
Root canal therapy	10 (50%)	13 (81%)	<0.052
Extraction	10 (50%)	3 (19%)	

^∗^Significant at *P* < 0.05.

## Data Availability

All the data have been included in the manuscript and are available on request to the corresponding author.
